# Comparative Genomic Analysis of Two *Xanthomonas oryzae* pv. *oryzae* Strains Isolated From Low Land and High Mountain Paddies in Guangxi, China

**DOI:** 10.3389/fmicb.2022.867633

**Published:** 2022-04-28

**Authors:** Tianjiao Li, Yiming Li, Xiuguo Ma, Xue Dan, Xianjiao Huang, Qinying Li, Shimin Lei, Zhengchun Zhang, Sheng Huang, Wei Jiang, Yanhua Yu, Yong-Qiang He

**Affiliations:** ^1^College of Agriculture, Guangxi University, Nanning, China; ^2^State Key Laboratory for Conservation and Utilization of Subtropical Agro-Bioresource and College of Life Science and Technology, Nanning, China; ^3^Napo Agricultural and Rural Bureau, Napo County, Baise, China; ^4^New Rural Development Institute of Guangxi University, Nanning, China

**Keywords:** *Xanthomonas oryzae* pv. *oryzae*, HiFi PacBio sequencing, genome, *tal*, high mountain paddy

## Abstract

*Xanthomonas oryzae* pv. textitoryzae (*Xoo*) is a causal agent of rice bacterial leaf blight (BLB), the major rice disease, which is seriously constraining rice production in Asia. The interaction between *Xoo* and rice is in a dynamic process, essentially the co-evolution. Tracking the occurrence of plant diseases and identifying the epidemic pathogens in time are critical to assessing the epidemic disease status and understanding the pathogen evolution. In 2020, the occurrences of rice BLB were spotted in many places of Guangxi, the major rice growing region in China. Two of the 2020-epidemic *Xoo* strains, namely, GXO20-01 and GXO20-06, were isolated from low land and high mountain paddies in Guangxi, respectively, and were demonstrated to be race R8 of Chinese *Xoo* strains, but with significantly different virulence on certain susceptible varieties of rice. The HiFi PacBio sequencing revealed that GXO20-01 and GXO20-06 share the highly syntenic genome structures and the major genome contents, but only differ in <10 genes, including one gene encoding for transcription activator-like effector (TALE). A phylogenomic analysis grouped GXO20-01 and GXO20-06 into the PX-A lineage, stood close to PXO563 and PXO71 strains, but stood away from the other Chinese *Xoo* strains; for example, the JL25 and YC11. A comparative genomic analysis revealed that the major pathogenicity/virulence genes are conserved in two, newly isolated *Xoo* strains and the other *Xoo* strains in PX-A lineage, including the majority genes for the TALomes. The genomic differences between the *Xoo* strains were pinpointed to a few *tal* genes, which were variable in both their numbers and sequences, even between GXO20-01 and GXO20-06, the two 2020-epidemic *Xoo* strains. The study further revealed the instability and variability of *tal* genes in *Xoo* and highlighted the utility of HiFi long-read sequencing in TALE analysis and pathogen tracking.

## Introduction

The gram-negative bacterium *Xanthomonas oryzae* pv. *oryzae* (*Xoo*) causes the rice bacterial leaf blight (BLB), one of the most destructive rice diseases in the major rice-growing regions worldwide, seriously affecting the quantity and quality of the staple crop rice (Niño-Liu et al., 2006). The interactions between *Xoo* and rice plants follow the gene for gene hypothesis to some extent, enabling *Xoo*-rice pathosystem as an excellent model platform in plant sciences (Zhang and Wang, 2013). The *Xoo* infects host plants depending on a series of virulence factors, including adhesins, degradative enzymes, polysaccharides, and especially the proteinous effectors, translocated *via* the type III secretion system, which is also known as type III secretion effectors (T3SEs) (An et al., 2020). Among the T3SEs, the transcription activator-like effectors (TALEs), which are presumed as one of the highest developed and most complex pathogenic factors in phytopathogenic bacteria, play crucial roles in pathogenicity and racial discriminations in *Xoo*-rice interaction (Erkes et al., 2017). A typical TALE consists of three parts: The *N*-terminal domain containing the type III secretion signal, the C-terminal domain that is acting a role in nuclear localization and transcriptional activation, and the central highly conserved repeat units. Each repeat unit contains 33–35 amino acids, in which the 12th and 13th amino acids are variable, called the repeat variable di-residue (RVD). Different RVDs can bind different bases of target DNA, thus ensuring that TALEs contain multiple RVDs that can recognize specific DNA sequences (Mak et al., 2012). The main function of a TALE is binding to the effector binding element (EBE) in the host genome to induce the expression of downstream susceptibility genes (Kay and Bonas, 2009; Moscou and Bogdanove, 2009). In most cases, the target genes are sugar–transporter genes, that is, the SWEET genes (Streubel et al., 2013). Alternatively, some TALEs, especially the iTALE, may trigger plant defense by activating an executor resistance gene or neutralizing R-gene-mediated plant disease resistance (Ji et al., 2016; Read et al., 2016).

The TALE-encoding genes (*tal* genes) in *Xoo* are presumed to be highly dynamic and variations in TALE repeats, such as recombination, duplication, InDels, and SNPs in RVDs, have greatly contributed to the production of new toxicity and the escape of plant immunity by the pathogens (Erkes et al., 2017). Tracking and identifying the variation of *tal* gene in the emerging or reemerging *Xoo* strains are helpful for accurate disease diagnosis and disease resistance breeding. However, the highly repetitive structure and the extreme similarity among the different members of *tal* genes in an individual *Xoo* strain greatly constrict correctly sequencing and assembling of such genes in the genome by using previous sequencing methods, e.g., the Sanger and the second-generation sequencing methods. Recently, long-read sequencing technologies are overcoming the early limitations in accuracy and throughput, thereby broadening their application domains in complex genomics. In GenBank, there are about 113 *Xoo* strains whose genomes were claimed to have been completely sequenced, but their sequencing qualities are of significant differences. The *tal* gene sequences and their distributions are of significant diversity amongst *Xoo* strains. An individual *Xoo* strain usually contains 9–21 *tal* genes, 9 per genome for African strains, and 13–21 per genome for Asian strains (Oliva et al., 2019). To date, about 100 *tal* genes composing the pan-TALome of *Xoo*, divided into 30 types, have been identified in *Xoo* strains. In rice plants, there are more than 20 OsSWEET genes, but in nature, only three of them, namely, OsSWEET11, OsSWEET13, and OsSWEET14 are demonstrated to be induced by *Xoo* (Oliva et al., 2019). In view of this, there are still a lot of unknown parts to be excavated in the updating *Xoo*-rice pathosystem.

In the time duration between the 1970s and 1990s, the rice BLB disease was rampant in the vast rice growing area, the south of the Yangtze River, which caused great losses to rice production in China. However, in the early 21st century, the rice BLB suddenly disappeared in most of the rice growing areas in China for some period of time, supposedly due to the breeding and promotion of disease-resistant rice varieties and the intensive applications of chemical germicides and antibiotics. However, in the last decade, the cases of BLB disease have been observed or/and reported increasingly in major rice growing areas in China, even serious occurrences in some certain areas were witnessed (Chen et al., 2012, 2016, 2017, 2019, 2021). The roller coaster pattern of the BLB incidences just reflects the dynamic zig-zag model in the interaction of pathogen and host plants while their long-term co-evolution in the virulence factors and the resistance genes (Jones and Dangl, 2006). Under certain environmental conditions, the occurrence of plant diseases indicates that the balance between plants and pathogens has been broken. The reason is either the loss of plant disease resistance genes or the evolution of virulence factors of pathogens. Therefore, the investigation and documentation of the occurrences or re-occurrences of *Xoo* strains were conducive to the effective control of BLB and targeted the disease-resistant breeding in rice production.

Guangxi is located in southern China at low latitude, which shares borders with Vietnam, and the Tropic of Cancer passes across its center. With a subtropical monsoon humid climate in Guangxi, the abundant rainfall and fairly high temperature ensure most of Guangxi is a highly suitable double cropping rice region in China. However, Guangxi has many mountainous areas where the lower accumulated temperature can only sustain the single-cropping rice production. The complex cropping rice production systems (such as mixed single- and double-cropping) pose potential challenges in plant protection. Previously, it was noticed that the differences in occurrences and incidences of rice bacterial blight in between high mountain and low land paddies in Guangxi, but little is known about the differences in their pathogens. In 2020, the occurrence of rice BLB was spotted in many places in Guangxi. We carried out a systematic investigation on rice BLB of Guangxi in 2020 and isolated the representative isolates from the typical disease area (Li et al., 2021). In this study, we focused on the comparative studies and genomic analysis on the representative *Xoo* strains of 2020 BLB occurrences isolated from low land and high mountain paddies in Guangxi, China.

## Materials and Methods

### Bacterial Strains and Rice Varieties Used in This Study

In 2020, we carried out a systematic survey about rice BLB in Guangxi (Li et al., 2021) and had isolated and identified the *Xoo* from the BLB frequent onset areas. The representative *Xoo* strains were named as GXO20-serial number; e.g., GXO20-01 in which GXO indicates the Guangxi *Xanthomonas oryzae* strain and 20-01 indicates chronologically the No. 1 strain isolated in 2020 (Table 1). To comparatively study *Xoo* strains isolated from the low land and the high mountain paddies in Guangxi, China, we have selected the representative *Xoo* strain GXO20-01 from Heng County, the typical low land area in Guangxi, and GXO20-06 from Napo County, the typical high mountain area in Guangxi. The distance between the two places is about 500 km, along the Tropic of Cancer. The bacterial strains used in this study are listed in Table 1. The *Xoo* international type strain *Xoo* PXO99^A^ was provided by the Institute of Plant Protection of Nanjing Agricultural University. The race R5 of Chinese strain YC11 was provided by the College of Life Science and Technology, Huazhong Agricultural University (Zheng et al., 2020).

**Table 1:** *Xanthomonas oryzae* pv. *oryzae* strains used in this study.

**Strains**	**Description**	**Collection location**	**Collection time**	**Altitude**	**Geographical position**	**Sources**
PXO99^A^	Type strain	The Philippines	1991	NA	NA	Salzberg et al., 2008
YC11	Reference strain, R5	Jiangsu, CHN	2014	NA	NA	Zheng et al., 2020
C9-3	*avrXa23* absent	Nanning, Guangxi, CHN	2017	NA	NA	Chen et al., 2021
GXO2001	Low land	Hengzhou, Guangxi, CHN	20200707	60 M ASL	22.70323/109.16997	This study
GXO2002	Low land	Laibin, Guangxi, CHN	20200810	90 M ASL	23.40913/108.98229	This study
GXO2004	High mountain	Napo, Guangxi, CHN	20200817	1200 M ASL	23.36471/105.78950	This study
GXO2006	High mountain	Napo, Guangxi, CHN	20200818	1200 M ASL	23.36428/105.79171	This study

The differential rice varieties used in this study were a simplified combination of near-isogenic lines (Fang et al., 1990; Liu et al., 2007) (Supplementary Table S1), IRBB2, IRBB3, IRBB5, IRBB13, IRBB14, and IR24, provided by the International Rice Research Institute (IRRI), Philippines (Ogawa et al., 1991), *via* the Institute of Plant Protection of Nanjing Agricultural University, China.

### Plant Assays and Pathotypic Analysis

The *Xoo* strains stored at −80°C were activated by streak culture on PSG medium (10 g l-1 peptone, 10 g l-1 sucrose, 1 g l-1 glutamic acid, pH 7.0) agar plates. The batch cultures of *Xoo* strains were grown in PSG medium for 18 h, about to the mid-log phase and then collected, and resuspended to the optical density at 600 nm (OD_600_) of 0.5.in sterile water.

The rice seeds were sown in plastic pots in a greenhouse under a 12-h light/dark cycle at 25–28°C, and the 45-day-old seedlings were clip-inoculated with a bacterial suspension of 0.5 OD_600_ and maintained under conditions of 80% relative humidity. Twenty leaves of each differential were inoculated with each isolate or sterilized water (as control). The disease severity was scored based on the visual assessment of the lesion length 14 days post-inoculation. The leaves with a lesion length of <1/4 and ≥1/4 were classified as resistant (R), and susceptible (S), respectively (Chen et al., 2019). All experiments were repeated three times. The least significant difference method (LSD method) was used for data analysis and the box-plot was generated using Origin 2018. A pathotypic analysis of *Xoo* strains was performed according to their reactions on NIL rice (Supplementary Table S1).

### Genome Sequencing and Assemblage

For the whole genome sequencing, *Xoo* strains were grown in PSG medium for 36 h, about to the early stationary phase, and then harvested. The genomic DNA of each strain was isolated and purified by using QIAGEN genomic-tip 20G following the manufacturer's protocol. The DNA samples were fragmented using g-TUBE. HiFi sequencing libraries were prepared using SMRTbell™ Express Template Prep Kit 2.0. The libraries were further size selected using BluePippin Systems. The average fragment length of the sequencing library is about 15 kb for circular consensus sequencing (CCS) to achieve long and high-fidelity (HiFi) reads (Tang, 2019). The DNA sequencing was performed using PacBio Sequel II System (Pacific Biosciences, USA) at Biomarker Technologies CO., LTD (Beijing, China). The HiFi reads have been *de novo* assembled using the Hifiasm software (Cheng et al., 2021). The Pilon v1.22 software was used to correct the assembly with Illumina data, and Circulator v1.5.5 software was used to cyclize and adjust the starting site. The genome completeness and contamination were evaluated by CheckM v1.1.3 (Parks et al., 2015).

### Genome Annotation and the Categorical Analysis

The preliminary genome annotation was carried out by NCBI prokaryotic genome annotation pipeline (Tatusova et al., 2016) and GeneMarkS-2 software (Lomsadze et al., 2018). The gene function and pathway analysis (Supplementary Table S2) were performed by BLASTP searches against functional databases, including Cluster of Orthologous Groups of proteins (COG) database (https://www.ncbi.nlm.nih.gov/COG/), Gene Ontology (GO) (http://geneontology.org/), and the Kyoto Encyclopedia of Genes and Genomes (KEGG) database, http://www.genome.jp/kegg/).

To decipher the *Xoo* genome from the phytopathological perspective, the confirmed and putative pathogenicity/virulence genes in *Xoo* were grouped into certain categories (Supplementary Table S3) in accordance with the genome categorical system for *Xanthomonas* first used by da Silva et al. (2002) and further improved by Thieme et al. (2005). The TAL effectors of each genome were identified and classified by AnnoTALE (Grau et al., 2016).

### Comparative Genomics and Phylogenomic Analysis

For structural comparison, complete genomes were aligned using progressive Mauve (Darling et al., 2010) with default settings. For phylogenomic analysis, complete genomes were aligned using Mauve. The tree was annotated and visualized by using the CVTree3 web server (http://cvtree.online/v3/cvtree/). The phylogenetic trees were constructed by using the neighbor-joining (NJ) method. The K-tuple length is 6 (Zuo and Hao, 2015). Orthologous genes of GXO20-01 and GXO20-06 were extracted from Mauve in pairwise comparison (Darling et al., 2010).

## Results

### Pathotyping of *Xoo* Strains From Low Land and High Mountain Paddies in Guangxi, China

To comparatively study the 2020-epidemic *Xoo* strains isolated from low land and high mountain paddies, we had chosen two *Xoo* strains, GXO20-01 and GXO20-02, from Heng County (the typical low land area) and two strains, GXO20-04 and GXO20-06, from Napo County (the typical high mountain area), respectively (Table 1). On the basis of molecular identification and back inoculation, the four *Xoo* strains were demonstrated to be quite similar in phenotypic characteristics, including EPS production, extracellular enzyme activity, motility, stress tolerance, and biofilm formation (data not shown).

**Figure 1:**
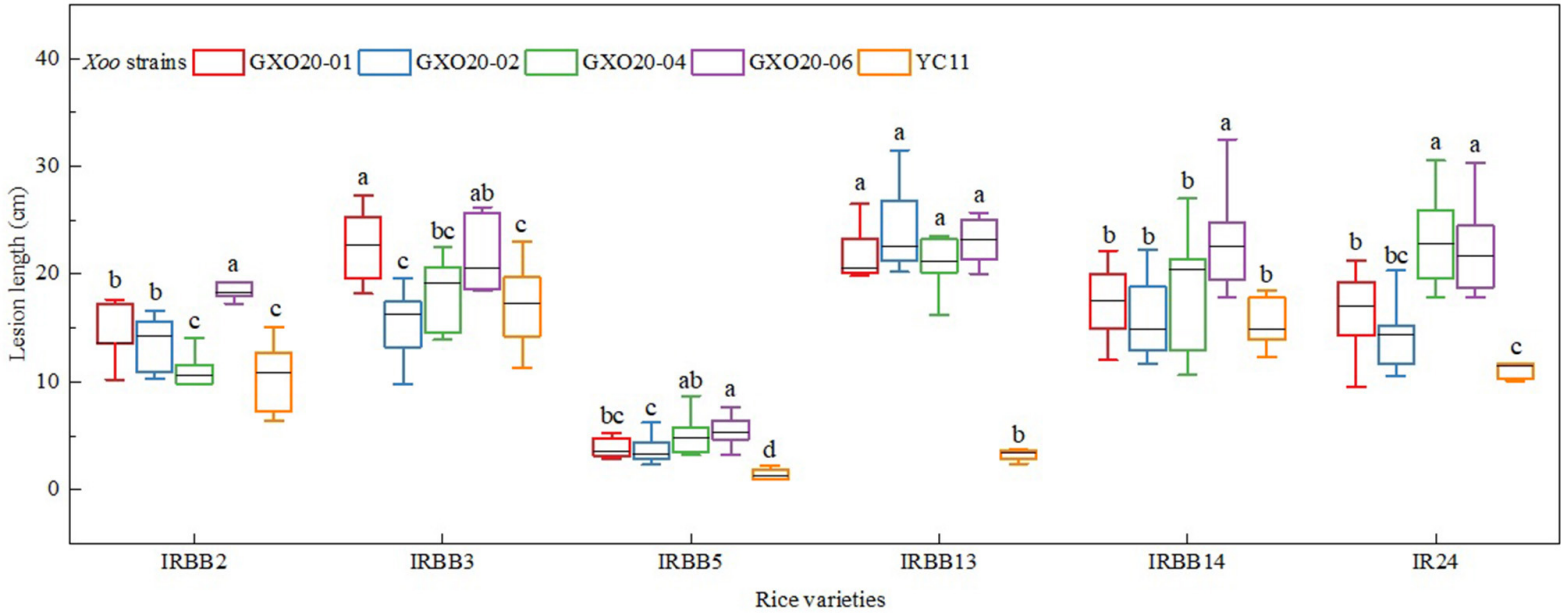
Responses of disease of newly isolated *Xoo* strains test on IRBB NILs. The least significant difference (LSD) test was used for data analysis at *P* < 0.05 level, showing as lowercase letters.

The pathotypic analysis of *Xoo* strains was commonly performed by using a simplified combination of IRBB NILs, IRBB2, IRBB3, IRBB5, IRBB13, IRBB14, and IR24, in China (Liu et al., 2007; Chen et al., 2012). In this study, the positive control *Xoo* strain YC11 clearly showed the SSRRSS interaction results on the differential rice varieties (Zheng et al., 2020). The pathotyping results demonstrated that all of four representative *Xoo* strains from low land and high mountain paddies in Guangxi belong to race R8, indicated by the SSRSSS interaction results (Figure 1). Additionally, statistical analysis showed that there were significant differences between those *Xoo* strains on virulence in some certain rice varieties (Figure 1).

### Complete Genome Characteristics of GXO20-01 and GXO20-06 Genomes

To quickly and effectively compare the genomic information of *Xoo* strains isolated from low land and high mountain paddies, GXO20-01 and GX20-06 were selected as the representative *Xoo* strains for low land and high mountain paddies, respectively (Table 1). Complete genome sequencing of two *Xoo* strains was performed using the Pacific Biosciences (PacBio) platform with high-fidelity (HiFi) long reads (see Materials and Methods). The CCS data for GXO20-01 and GX20-06 are as follows: SeqNum: 6357 and 5238 reads, SumBase: 6,106,727 and 53,252,594 bp, N50 length: 10,505 and 10,286 bp. The HiFi reads have been *de novo* assembled using the Hifiasm software (Cheng et al., 2021). The Pilon v1.22 software was used to correct the assembly with Illumina data, and Circulator v1.5.5 software was used to cyclize and adjust the starting site. One contig was assembled for each genome, with sizes 4,963,479 and 4,962,861 bp, respectively (Table 2). Two *Xoo* strains contain no plasmid. The DNA completeness for the two genomes was found to be 99.62 and 99.44%, respectively, and contamination for the two genomes was 0.37%. The basic genome features of GXO20-01 and GX20-06 and other sequenced *Xoo* strains were listed in Table 2. Also, the basic genome features of GXO20-01 and GX20-06 were annotated to encode 724 and 727 pseudogenes, similar to those found in PXO99^A^, 759 (Salzberg et al., 2008) and in MAFF 311018, 747 (Ochiai et al., 2005), respectively (Table 2). These results also indicated that the HiFi sequencing quality is relatively high.

**Table 2:** The genomic features of *Xoo* GXO20-01 and GXO20-06 and other *Xoo* strains.

**Genome features**	**GXO20-01**	**GXO20-06**	**PXO99^**A**^**	**PXO99^**A**^-GX**	**PXO86**	**BXO1**	**IXO1088**	**KACC 10331**	**MAFF 311018**	**YC11**	**LN4**	**C9-3**
Genome size (bp)	4,963,479	4,962,861	5,238,555	5,028,051	5,016,623	4,991,257	5,093,052	4,941,439	4,940,217	4,867,200	5,012,583	4,924,298
G+C content (%)	63.70	63.71	63.60	63.65	63.72	63.63	63.67	63.69	63.70	63.74	63.60	63.70
Coding density (%)	86.71	86.71	86.70	86.45	86.79	87.84	86.49	87.81	86.76	86.68	86.66	86.73
Genes	4,672	4,666	4,877	4,699	4,672	5,076	4,758	4,939	4,644	4,540	5,004	4,616
CDSs	4,468	4,462	4,666	4,493	4,470	4,873	4,554	4,734	4,438	4,335	4,800	4,412
tRNA	53	53	54	53	53	53	53	53	53	53	53	53
rRNA	6	6	6	6	6	6	6	6	6	6	6	6
ncRNA	145	145	151	147	143	144	145	146	147	146	145	145
TALE genes	17	18	19	17	18	18	19	13	17	12	17	17
Average CDS length	947	946	957	953	957	897	950	911	960	967	893	952
Pseudogenes	724	727	759	737	720	987	745	1,013	747	702	3,293	718
Plasmid	0	0	0	0	0	2	0	0	0	0	0	0

### Genome Annotation and the Categorical Analysis of Pathogenicity/Virulence Genes

The genomes were annotated 4,468 and 4,462 protein encoding sequences (CDSs) in GXO20-01 and GXO20-06, respectively (Table 2; Supplementary Table S2), among which 628 and 629 in GXO20-01 and GXO20-06, respectively (Supplementary Table S3), were annotated as pathogenicity/virulence genes in accordance with the genome categorical system for *Xanthomonas* firstly used by da Silva et al. (2002) and further improved by Thieme et al. (2005). The *Xoo* genomes encode typical pathogenicity/virulence factors found in *Xanthomonas* spp., including extracellular hydrolases, extracellular polysaccharides, adhesins, type II secretion system, type III secretion system, and T3SEs (White et al., 2009; An et al., 2020).

In this study, the pathogenicity-related genes in *Xoo* were divided into 10 groups: (1) Type III secretion system (*hrp*/*hrc* genes) and its effectors, (2) other secretion systems, (3) extracellular enzymes, (4) surface polysaccharides, lipopolysaccharides, and antigens, (5) toxin and detoxification, (6) adhesion and biofilm, (7) quorum sensing and regulation of virulence factors, (8) adaptation to atypical conditions, (9) bacterial motility, and (10) other factors in bacterium–plant interaction (Supplementary Table S3). The detailed genome annotation might be helpful to elucidate the pathogenesis of this typical plant pathogenic bacterium and to pinpoint the certain genes in different infection stages (Supplementary Table S3).

### Genome Structures and Genome Contents for GXO20-01 and GXO20-06 Are Strikingly Similar

**Figure 2:**
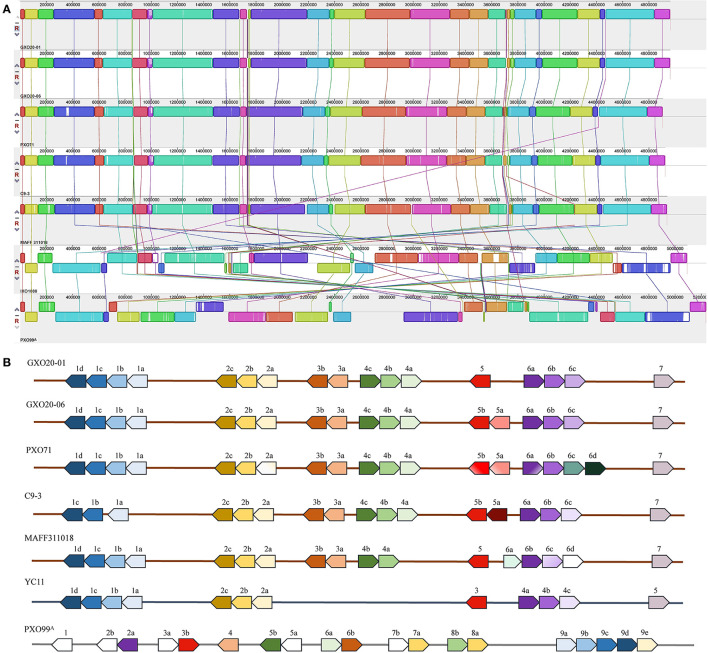
Comparison of whole genomes and *tal* genes of *Xoo* strains. **(A)** Progressive Mauve alignments chromosomes of *Xoo* strains. Colinear blocks are shown in green, with gray indicating regions of sequence dissimilarity. The ruler indicates distance from the annotated origin in base pairs. **(B)** The *tal* genes of *Xoo* strains. The genes are represented as arrows at their relative positions in the linearized chromosome. Arabic numerals indicate the serial number of *tal* gene clusters in an *Xoo* strain, and each lowercase letter indicates each *tal* gene in a certain gene cluster.

To clarify the relationship between the two strains, we have conducted pairwise comparative analysis both in their genome structures and genome contents. The results showed that the chromosomes of GXO20-01 and GXO20-06 are entirely syntenous (Figure 2A). The positions of the 17 allelic *tal* genes show no duplications or rearrangements in one genome relative to the other (Figure 2B). The results of genome two-way alignments indicated that the two genomes are conserved both in gene contents and their allelic sites (Supplementary Table S2). Among the 4,468 and 4,462 annotated genes in GXO20-01 and GXO20-06, 4,442 genes are orthologous. Totally, there are 123 genomic variational sites between genomes, including 97 SNPs, 19 indels, and 7 complex variations. Among the SNPs, 76 were found in coding sequence genes, 33 SNPs were missense variants that resulted in 20 gene differences between two strains, as more than one SNPs in one single gene. The indels and complex variations also caused nine gene differences between the two strains (Table 3; Supplementary Tables S2, S3). Here, GXO20-01 has 6 strain-specific genes encoding a 5-amino-6-(5-phosphoribosylamino) uracil reductase (RibD), a GNAT family *N*-acetyltransferase, two adenylyl–sulfate kinase, outer membrane protein, and a hypothetical protein, and GXO20-06 has only one strain-specific gene, the tal5a encoding a TalDR-like TALE. Subsequently, it was found that the two TALE repertoires are nearly identical but only one TALE missing in GXO20-01. Consequently, the pairwise genomic comparison and phylogenetic analysis suggested that GXO20-01 and GXO20-06 are probably sibling *Xoo* strains.

**Table 3:** The genomic variations and differential genes between *Xoo* strains GXO20-01 and GXO20-06.

**Type of genomic variations**			**Gnomic variation events**	**Differential genes**	**GXO20-01**	**GXO20-06**
SNPs	Intergenic region		21	0		
	In genes	synonymous variant	43	0		
		missense variant	32	19	10	9
		stop gained	1	1		1
insertions		Intergenic region	8	0		
		In genes	1	1		
deletions		Intergenic region	5	0		
		In genes	5	5	3	2
complex variations		Intergenic region	3	0		
		In genes	4	3	2	1
Total			123	29	15	14

### The Relationships Analysis of the Two Guangxi Strains With Other *Xoo* Strains

Multiple alignments of the selected *Xoo* genomes showed that the genome structures of GXO20-01 and GXO20-06 are highly collinear with *Xoo* strains in lineage PX-A (Quibod et al., 2016) (Figure 2A) and the loci of the major *tal* genes are highly conserved (Figure 2B).

**Figure 3:**
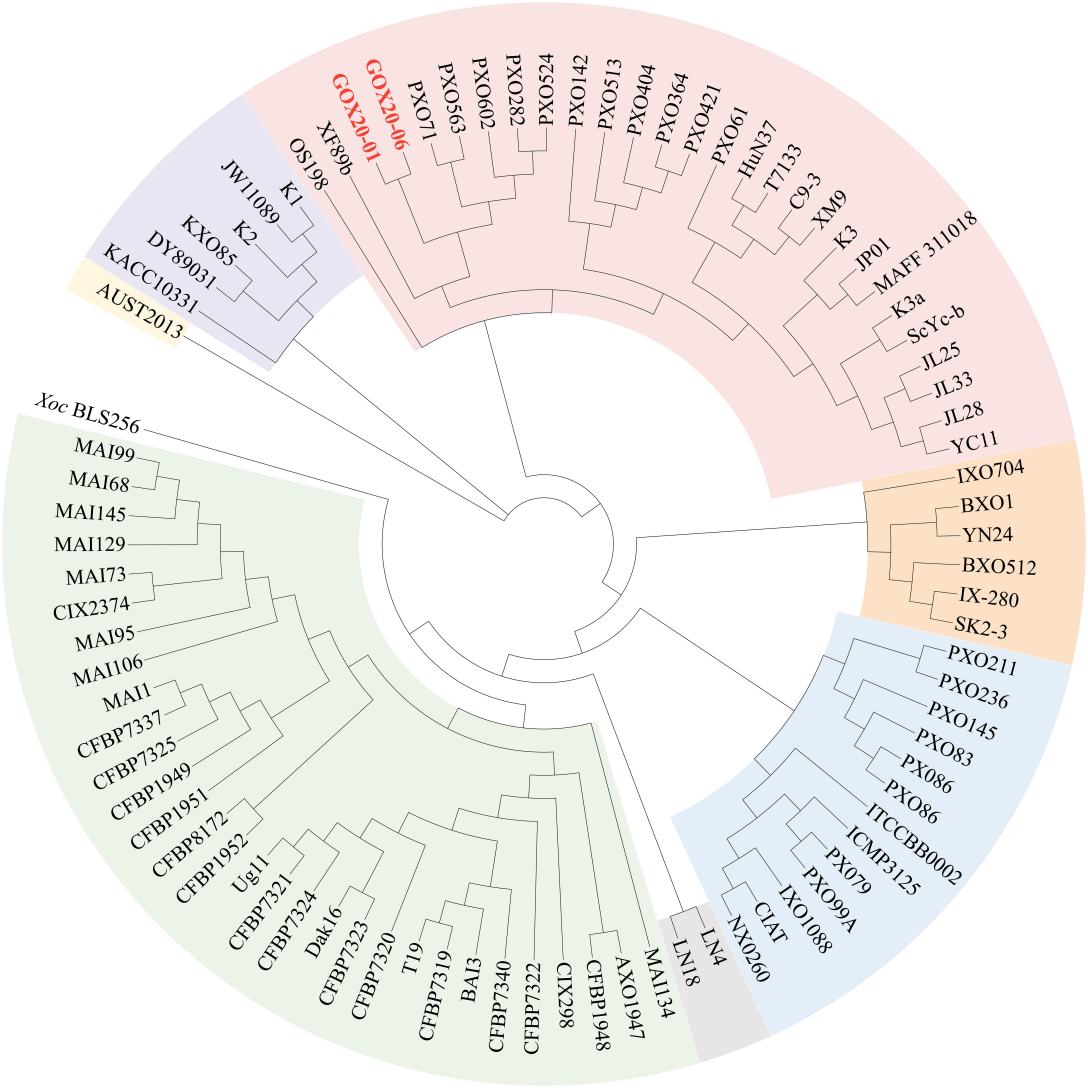
Phylogenomic analysis of *Xoo* strains based on genome sequences of representative *Xoo* strains. The genome sequences were obtained from NCBI. The tree was annotated and visualized by using CVTree3 web server. The phylogenetic trees by using the NJ method. The K-tuple length is 6.

To further determine the relationships of the newly sequenced *Xoo* strains with other *Xoo* strains, we carried out a phylogenomic analysis in which representative *Xoo* strains were selected representing typical *Xoo* strains from around the world (Figure 3). The phylogenomic analysis clearly grouped GXO20-01 and GXO20-06 in the branch of PXO563 and XF89b in lineage PX-A (Quibod et al., 2016) or Xoo-A (Chien et al., 2019), but away from the Chinese mainland strains (Zheng et al., 2020).

### The GXO20-06 *Tal5a* Gene Situated in the Variable *tal* Locus Encodes a TalDR-Like TALE

The TALEs are the most important virulence factor used by *Xoo* to interact with rice, enforcing virulence, proliferation, and dissemination against rice (Erkes et al., 2017). AnnoTALE analysis indicated that GXO20-01 and GXO20-06 contain 17 and 18 *tal* genes, respectively. Among the 17 shared TALEs, 15 typical TALEs are assigned to classes TalAF, TalAN, TalAD, TalAB, TalAL, TalAD, TalAO, TalAE, TalAM, TalAH, TalAA, TalAR, TalAG, TalAS, TalAQ, and the iTALE/truncTALEs are assigned to class TalAI. Compared with the closely related *Xoo* strains, GXO20-01 and GXO20-06 contain no TalFX that is found in C9-3, no TalBK that is found in LN4, and no TalDW that is found in XF89b (Table 4).

**Table 4:** RVD sequences in TALEs of *Xoo* strains GXO20-01 and GXO20-06.

**TALE class^a^**	***Xoo* strain**	**RVD sequence**
TalAE	GXO20-01; GXO20-06; C9-3	NI NN NI HG HD NG HD HG HD HD HD NG
	MAFF311018; PXO99^A^ (*tal9d*); YC11	NI NN NI HG HG NV HG HD HG HD HD HD NG
TalAO	GXO20-01; GXO20-06; C9-3	NI NN N* NG NS NN NN NN NI NN NI NG HD HD NI NG –
	MAFF311018; PXO99^A^ (*tal9c*/*avrXa27*); YC11	NI NN N* NG NS NN NN NN NI NN NI N* HD HD NI NG NG
TalAQ	GXO20-01; GXO20-06	HD HD NN NN NI NG HD S* HG HD NG N* NG HD HD N* NI NI NN HD HI ND HD NG NN HG N*
	MAFF311018; YC11	HD HD NN NN NS NG HD S* HG HD NG N* HD HD HD N* NN NI NN HD HI ND HD HG NN HG N*
	PXO99^A^ (*tal9b*)	HD HD NN NN NG NG HD NS HG HD NG N* HD HD HD N* NN NI NN HD HI ND HD HG NN HG N*
TalAP	GXO20-01; GXO20-06; C9-3; PXO99^A^ (*tal9a*/*pthXo8*)	HD HD HD NG N* NN HD HD N* NI NI NN HD HI ND HD NI HD NG NG
	MAFF311018; YC11	HD HD HD NG N* NG HD S* HG NI NI NN HD NN ND HD NI HD HG NG
TalAL	GXO20-01; GXO20-06; C9-3	NI NS HD NG NS NN HD N* NN NN NI NN HD HG HD HD NN NG
	MAFF311018; YC11	NI NS HD NG NS NN HD N* NN NN NI NG HD NG HD HD HD NG
TalAB	GXO20-01; GXO20-06; C9-3; PXO99^A^ (*tal7a, tal8a*)	NI HG NI NI NI NN HD NS NN NS NN HD NN NI HD NN NS NG – –
	MAFF311018; YC11	NI HG NI NI NI NN HD NS NN NS NN HD NN NI HD NN NI NG HD NG
TalAD	GXO20-01; GXO20-06; MAFF311018; C9-3; PXO99^A^ (*tal9e*)	NN HD NS NG HD NN N* NI HD NS HD NN HD NN HD NN NN NN NN NN NN NN HD NG
	YC11	NN HD NS NG HD NN N* NN HD NS HD NN HD NN HD NN NN NN NN NN NN NN HD NG
TalAN	GXO20-01; GXO20-06; C9-3	NI N* NI HG NI NI NS HD NN HD NS NG SS HD NI NI NN NI NN NI NG
	MAFF311018	NI N* NI HG NI NI NS HD NN HD NS NG SS HD NI NI NN NI NN NS NG
	PXO99^A^ (*tal6b*)	NI HG NI HG NI NI NI HD NN HD NS NG SS HD NI NI NN NI NN NI NG
TalAF	GXO20-01; GXO20-06; MAFF311018; C9-3; PXO99^A^ (*tal4*)	NI NN NN NI NI NI HD NS HG NN NN NN NI NI NG HD
TalAH	GXO20-01; GXO20-06; MAFF311018; C9-3	NI N* NI NS NN NG NN NS N* NS NN NS N* HD HG HD NI HD HD NG
	PXO99^A^ (*tal6a*)	NI N* NI NS NN NG NN NS N* NS NN NS N* NI HG HD NI HD HD NG
TalAA	GXO20-01; GXO20-06; C9-3	NI HG NI NG HG HD NS NG HD NN NG HG NG HD HG HD HD NI NN NG
	PXO99^A^(*tal8b*)	NI HG NS HG HG HD NS NG HD NN NG HG NG HD HG HD HD NI NN NG
	MAFF311018	NI HG NI NG HG HD NS NG HD NN NG HG NG HD HG HD HD NI NS HG HD NI N* NS NI NI HD HD N* NS N*
TalAR	GXO20-01; GXO20-06; C9-3	NI H* NI NN NN NN NN NN HD NI NS HG HD NI N* NS NI NI HD HD N* NS N*
	PXO99^A^ (*tal5b*/*pthXo6*)	NI H* NI NN NN NN NN NN HD NI HD HG HD NI N* NS NI NI HG HD NS NS NG
TalAI	GXO20-01; GXO20-06; MAFF311018; C9-3; YC11	NS HD NG NG NG NG HD HD HD HD NN HD NG HD NI HD NN N*
	PXO99^A^ (*tal3b*)	NS HD NG NG NG NG NG HD HD HD NN HD NG HD HD HD HD N*
**TalDR-like**	**GXO20-06**	NS HD NG NG NG HD HD HD HD NN HD HD HD HD NN H*
TalAG	GXO20-01; GXO20-06; MAFF311018; C9-3; YC11	NI NG NN NG NK NG NI NN NI NN NI NN NS NG NS NN NI N* NS NG
	PXO99^A^ (*tal2a*)	NI NG NN NG NK NG NI NN NI NN NI – – – – – HD N* NS N*
TalAS	GXO20-01; GXO20-06	NI HG NI HD NI HD NN HD HD HD NI NI NN NI HD HD HD HG NN NN HD NS NN HD N* NS N*
	MAFF311018; YC11	NI HG NI NI HG HD NN HD HD HD NI NI NN NI HD HD HD HG NN NN HD NS NN HD NG NS N*
TalDV	GXO20-01; GXO20-06	NI HG NI NI NS HD NN HD HD HD NS HD N* NI HD HD NN NS NN NN NG NN HD N* NS NS NS N*
TalAM	GXO20-01; GXO20-06; MAFF311018; C9-3; YC11	NI HG NI NN NN NI NN HD NI HD NS NS NS HD NN HD NG HD HD HD NG NG

a The TALE classes based on AnnoTALE (Grau et al., 2016). The “*” value indicates the absent of an amino acid residue in certain RVDs. The colour letters highlight the different RVDs in between Xoo strains. The bold values highlight the unique TAL gene in the newly sequenced Xoo strain GXO20-06.

The diversity in TALE repertoires of *Xoo* actually underpins the complexity of the virulence of *Xoo* races. A general survey of *Xoo* genomes in GenBank indicated that there are not two *Xoo* strains sharing the identical TALome. In our case, the closely related sibling *Xoo* strains GXO20-01 and GXO20-06 still have one TALE gene difference.

**Figure 4:**
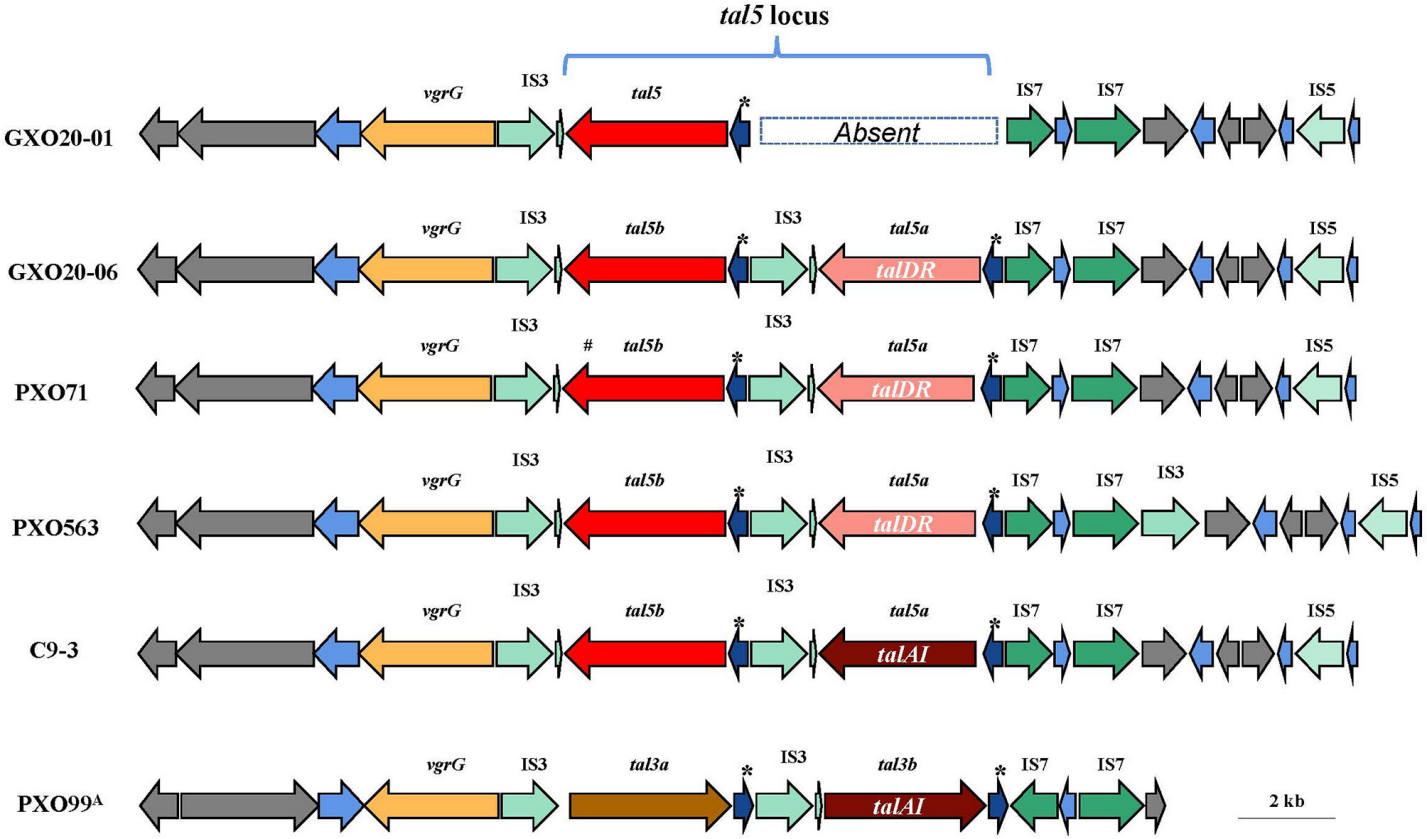
Comparison of the genomic location that contains the *tal5* gene cluster. Genes are depicted by arrows indicating the transcription direction. The “*” indicates the direct repeat sequences. *tal5a* of C9-3 is a truncated *tal* gene encoding a TalAI effector.

It is worth mentioning that GXO20-06 contains a *tal5a* gene situated in the variable *tal* locus that encodes a TalDR-like TALE, which is missing in GXO20-01. TalDR is a particular TALE that is found only in the PXO563 and PXO71. The *tal5* locus (here referred to as GXO20-06 genome) contains a number of insertion sequences and multiple repeat sequences (Figure 4), which is considered to be the most unstable section in *Xoo* genomes. The genomic comparison indicated that in most *Xoo* genomes, *tal5* locus or its homologous sections encode one or two iTALE/truncTALEs, but their CDS sequences differ largely (Table 4). In most cases, *talDR/talDR-like* gene totally disappeared in certain *Xoo* genomes. Such TALE diversities might provide a novel marker or reference for the genotyping or tracking of pathogens. Also, GXO20-06 is isolated from high mountain paddies, which still carry the *talDR/talDR-like* gene in the chromosome, suggesting the slow variation probably due to mild selection pressure from a single cropping rice system. As epidemic *Xoo* strains, it is expected GXO20-01 and GXO20-06 strains with only one TALE distinct to be the ideal reference strains in the research on the pathogenesis, virulence evolution, and pathogen tracking of rice BLB in China and even in many regions worldwide.

## Discussion

Guangxi is the major rice producing region in China and is presumed to be one of the central areas of ancient rice domestication (Huang et al., 2012). Guangxi is also known as one of the hardest hit areas of rice diseases in China (Lin et al., 2021). In 2020, we have carried out a systematic survey on rice BLB in Guangxi. A batch of epidemic *Xoo* strains was isolated and characterized. In this study, we have sequenced two *Xoo* strains GXO20-01 and GXO20-06, isolated from low land and high mountain paddies in Guangxi, respectively. Phylogenomic analysis and genome comparison indicated that GXO20-01 and GXO20-06 are the two *Xoo* sibling strains, with only differing few genes, including the *talDR/talDR-like* gene that was only found in PXO563 and PXO71, the Philippines strain (Quibod et al., 2016). As the *talDR/talDR-like* gene is absent from other Chinese *Xoo* strains, the relationships between the GXO20-06 and these two Philippines strains are interesting. As rice BLB is a frequent disease in many countries, it is helpful for effective prevention and control of the disease to establish multiple ways for diagnosing and tracking the virulence variations, e.g., the TALE typing.

The two strains GXO20-01 and GXO20-06 were chronologically isolated from low land and high mountain paddies in Guangxi, respectively. GXO20-01 is earlier than GXO20-06 to be collected from the paddies, however, GXO20-06 is assumed to be closer to their common ancestor according to their TALE repertoires. In view of the genome structure in the *tal5* locus, GXO20-01 might easily lose the *talDR/talDR-like* gene, other than GXO20-06 acquired the gene. Taking together the climates and cropping systems, it is tempting to conclude that the genomic variation pace of *Xoo* GXO20-06 in high mountain paddy is slower than that of GXO20-01 in low land paddy.

Pathotyping analysis showed that both GXO20-01 and GXO20-06 belong to the race R8 group which was demonstrated to be the predominant strain in the paddies of South China (Chen et al., 2017, 2019). It was shown that GXO20-01 and GXO20-06 cannot overcome the resistance of *xa5*, indicating that rice materials or varieties carrying the recessive *xa5* gene are of favorable superiority and wide application prospect in controlling rice BLB in Guangxi.

The TALEs are found only in a small number of bacterial species are believed to be one of the most complex virulence factors both in their molecular structures and their functional mechanisms (Erkes et al., 2017). With nearly identical, tandem-repeat-filled sequences in a single *tal* gene and with the multiple *tal* gene copies scattered in *Xanthomonas* genome, sequencing the genome of a *Xanthomonas oryzea* can be challenging (Booher et al., 2015). Elucidating *Xo* genomes require sequencing data that is both accurate and complete. Single Molecule, Real-Time (SMRT) Sequencing technology has evolved to a different type of long read, known as highly accurate long reads, or HiFi reads (Tang, 2019). Although the CCS read numbers, total sequencing bases and sequencing depth are significantly reduced, the PacBio® HiFi sequencing method yields highly accurate long-read sequencing datasets with read lengths averaging 10–25 kb and accuracies >99.5% (Tang, 2019; Cheng et al., 2021). In this study, the HiFi sequencing allowed quickly, effectively, and accurately assembling the complete gap-free genomes of *Xoo* strains.

In conclusion, by the utility of HiFi long-read sequencing in *Xoo* genome sequencing, this study further revealed the instability and variability of *tal* genes in the epidemic *Xoo* strains from different rice paddies. The approach and genome data provide information for discovering the new virulence factors and elucidating the functional mechanisms of TALEs in the *Xoo*-rice pathosystem.

## Data Availability Statement

The datasets presented in this study can be found in online repositories. The names of the repository/repositories and accession number(s) can be found in the article/Supplementary Material.

## Author Contributions

Y-QH and TL designed the research and drafted the manuscript. TL, XH, QL, and ZZ isolated the bacterial strains. TL, XD, and Y-QH isolated the genome DNA and performed the genomic data analysis. TL, XM, and SL conducted the plant assays and pathotyping. YL, SH, and WJ conducted bioinformatic analysis. WJ and YY contributed to revising the manuscript. All authors made their contributions to the manuscript, contributed to the interpretation of data, and approved the final manuscript.

## Funding

This work was supported by the National Key R&D Program of China (2018YFD0200302), the Natural Science Foundation of Guangxi (2019GXNSFAA245052), and the Graduate Education Innovation Fund Project in Guangxi (YCBZ2020016).

## Conflict of Interest

The authors declare that the research was conducted in the absence of any commercial or financial relationships that could be construed as a potential conflict of interest.

## Publisher's Note

All claims expressed in this article are solely those of the authors and do not necessarily represent those of their affiliated organizations, or those of the publisher, the editors and the reviewers. Any product that may be evaluated in this article, or claim that may be made by its manufacturer, is not guaranteed or endorsed by the publisher.
