# Supplementary material for: Comparative Genomic Analysis of Two Xanthomonas oryzae pv. oryzae Strains Isolated From Low Land and High Mountain Paddies in Guangxi, China
Source: Front Microbiol. 2022 Apr 28;13:867633. doi: 10.3389/fmicb.2022.867633 (PMC9096941; doi:10.3389/fmicb.2022.867633)
Supplement: Supplementary file 1 [file Table_1.DOCX]

**Table S1 The pathogenic reactions of *Xoo* strains on the differential hosts**

| **Race** | **Differential Rice Variety** | | | | | |
| --- | --- | --- | --- | --- | --- | --- |
|  | IRBB2  (*Xa2*) | IRBB3  (*Xa3*) | IRBB5  (*xa5*) | IRBB13  (*xa13*) | IRBB14  (*Xa14*) | IR24  (*Xa18**) |
| R1 | R | R | R | R | R | R |
| R2 | R | R | R | R | R | S |
| R3 | S | R | R | R | R | S |
| R4 | S | R | R | R | S | S |
| R5 | S | S | R | R | S | S |
| R6 | R | R | R | S | R | R |
| R7 | R | S | R | S | R | S |
| R8 | S | S | R | S | S | S |
| R9 | S | S | S | S | S | S |
| R10 | R | R | R | R | S | S |

R: Resistant; S: Susceptible. *Recurrent parent *Xa18*.
